# A preliminary study of a novel emergency department nursing triage simulation for research applications

**DOI:** 10.1186/s13104-016-2337-3

**Published:** 2017-01-03

**Authors:** Steven L. Dubovsky, Daniel Antonius, David G. Ellis, Werner Ceusters, Robert C. Sugarman, Renee Roberts, Sevie Kandifer, James Phillips, Elsa C. Daurignac, Kenneth E. Leonard, Lisa D. Butler, Jessica P. Castner, G. Richard Braen

**Affiliations:** 1 Department of Psychiatry, University at Buffalo, 462 Grider St, Room 1182, Buffalo, NY 14215 USA; 2 Departments of Psychiatry and Medicine, University of Colorado, Aurora, CO USA; 3 Department of Emergency Medicine, University at Buffalo, 462 Grider St, Buffalo, NY 14215 USA; 4 Department of Biomedical Informatics, University at Buffalo, 701 Ellicott St, Buffalo, NY 14203 USA; 5 School of Dental Medicine, University at Buffalo, 462 Grider St, Buffalo, NY 14215 USA; 6 Full Circle Studios, 710 Main St, Buffalo, NY 14202 USA; 7 Research Institute ON Addictions, University at Buffalo, 1021 Main St, Buffalo, NY 14203 USA; 8 School of Social Work, University at Buffalo, 685 Baldy Hall, Buffalo, NY USA; 9 School of Nursing, University at Buffalo, 212 Wende Hall, Buffalo, NY USA; 10 462 Grider St, Buffalo, NY 14215 USA; 11 4455 Genesee St, Buffalo, NY 14225 USA; 12 100 High St, Buffalo, NY USA

**Keywords:** Emergency department, Simulation, Computer, Disaster

## Abstract

**Background:**

Studying the effect on functioning of the emergency department of disasters with a potential impact on staff members themselves usually involves table top and simulated patient exercises. Computerized virtual reality simulations have the potential to configure a variety of scenarios to determine likely staff responses and how to address them without intensive utilization of resources. To decide whether such studies are justified, we determined whether a novel computer simulation has the potential to serve as a valid and reliable model of on essential function in a busy ED.

**Methods:**

Ten experienced female ED triage nurses (mean age 51) mastered navigating a virtual reality model of triage of 4 patients in an ED with which they were familiar, after which they were presented in a testing session with triage of 6 patients whose cases were developed using the Emergency Severity Index to represent a range of severity and complexity. Attitudes toward the simulation, and perceived workload in the simulation and on the job, were assessed with questionnaires and the NASA task load index. Z-scores were calculated for data points reflecting subject actions, the time to perform them, patient prioritization according to severity, and the importance of the tasks. Data from questionnaires and scales were analyzed with descriptive statistics and paired t tests using SPSS v. 21. Microsoft Excel was used to compute a correlation matrix for all standardized variables and all simulation data.

**Results:**

Nurses perceived their work on the simulation task to be equivalent to their workload on the job in all aspects except for physical exertion. Although they were able to work with written communications with the patients, verbal communication would have been preferable. Consistent with the workplace, variability in performance during triage reflected subject skill and experience and was correlated with comfort with the task. Time to perform triage corresponded to the time required in the ED and virtual patients were prioritized appropriately according to severity.

**Conclusions:**

This computerized simulation appears to be a reasonable accurate proxy for ED triage. If future studies of this kind of simulation with a broader range of subjects that includes verbal communication between virtual patients and subjects and interactions of multiple subjects, supports the initial impressions, the virtual ED could be used to study the impact of disaster scenarios on staff functioning.

**Electronic supplementary material:**

The online version of this article (doi:10.1186/s13104-016-2337-3) contains supplementary material, which is available to authorized users.

## Background

An essential component of the emergency department (ED) is to respond to disasters, infectious disease threats, and other extreme events. Responses to such events are increasingly hampered by increased visits and crowding in the face of decreasing numbers of EDs, beds and providers [1–3], among other factors. The impact of these global stresses is exacerbated when ED personnel are themselves at risk, as occurs with infectious diseases, especially during patient triage in the ED, before the patient is in isolation and appropriate personal protective equipment has been employed. To reduce this risk, hospitals have implemented rigorous infection control procedures that are followed to varying degrees [4].

In addition to personal risk, when an epidemic, earthquake, or other disaster threatens the homes and families of ED staff, it can affect their ability to cope with increased patient loads, their adherence to infectious disease protocols, and even their willingness to come to work [5, 6]. However, information about staff functioning during such events comes only from uncontrolled experience at the few sites at which the events have occurred. In order to determine the likely impact of unusual but potentially disastrous circumstances in order to to modify ED protocols accordingly, it would be helpful to develop simulated models of the ED that can be manipulated experimentally.

Computer simulations provide a tool for enhancing emergency preparedness by creating realistic visual representations of the various patient care challenges faced by emergency providers [7, 8]. Computer simulation is preferable to tabletop, mannequin and simulated live patient protocols because of decreased expense, lack of need to commit physical resources, ability to participate from off-site locations, and ease of reconfiguring a virtual ED to match the circumstance studied. In addition, virtual simulations can model the likely impact of different interventions without disrupting ongoing ED patient care [2, 9–11].

The most frequently used computerized ED model of emergency department patient flow is discrete event simulation (DES) [10], which is used to predict the effects of operational changes on patient throughput, waiting times, efficiency, length of stay, resource utilization and interaction of processes within a system [10, 12, 13]. An extension of DES is agent based modeling (ABM), which models behavior and its outcomes at the individual level [10]. A model using novel software to create a hierarchy of heterogeneous pseudo-agents has been used to represent patients moving through the emergency department during triage, evaluation by a physician, diagnostics, and treatment [10]. The main use of this model has been to develop optimal staffing models for different patient populations.

These computer simulations often focus on a specific factor, but addressing multiple systems that are impacted at the same time may be more realistic [14]. Virtual reality is a computerized model that expands the ability to model multiple influences on interactions of healthcare workers with each other, with patients, and with their environment. In a virtual reality simulation, virtual representations for patients, healthcare workers and other individuals may be automated (robots or “bots”), or they may be actively directed by the actual person they represent, in which case they are avatars. Avatars may then interact with each other and with robots. Second Life is an open-access, multi-user, virtual environment that has been used to train students in various fields [9] and to model multiple casualties in the field and in an emergency department for training [15].

GaMeTT, which has been used for training a military emergency response group, is a 3D, interactive, avatar-based simulation designed to train on an internet platform, that increases a sense of involvement (presence) by participants [16]. Arrow keys and the mouse control avatar movements. Using this model, an online virtual reality model of an emergency room was populated with 10 virtual patients exposed to radiation and 10 exposed to a toxin [17]. Of 10 physicians and 12 nurses participating in the training, 2/3 felt immersed in the virtual model all or most of the time. After the training, the percentage of subjects who felt confident or very confident in managing these events increased from 18 to 86%, with the majority attributing improved confidence to the training.

Since computer simulations have largely been used for training, the degree to which they can be used in a research setting remains to be determined. Other than a single simulation used to test the effect of different numbers of staff on patient flow [1], studies of the effectiveness of computer simulation in predicting outcomes such as the impact on the ED and its staff of epidemics and other disasters that alter patient flow and composition are lacking. Using photographs of our primary emergency department and actual patient scenarios from our practice, we adapted CliniSpace, a novel virtual reality platform used primarily for training for emergency management of trauma, that has a larger range of interactive bots and avatars than have been used previously [18], to develop a model of an ED that could be used to empirically study the possible impacts of such events. Because performance on this (or any other) simulation has not been compared with the actual situations it represents, it was necessary to demonstrate that it could be used as a valid model of an important component of ED activity before we could investigate the effect of varying parameters that impact it. We chose the discrete task of patient triage because it could be readily compared to performance at the actual site, and because most nursing staff who perform triage also work in other ED activities.

## Methods

### Ethics approval and consent to participate

This study was approved by the University at Buffalo Health Sciences Institutional Review Board. Written informed consent was obtained from 10 Caucasian female ED nurses with a mean age of 51.1 years (range: 34–63). Subjects were recruited through fliers in two local hospitals, announcements at meetings of the local Emergency Nurses Association, and word-of-mouth. All subjects were currently working full- or part-time performing ED triage. Demographic data, nursing experience, and experience with video gaming and virtual reality, are summarized in Table 1.

**Table 1:** Nursing and gaming experience

Highest education	
Associate’s degree in nursing	2
Bachelor of Science in nursing	7
Master of Science in nursing	1
Nursing experience, months (mean/SD)	303.5 (154.2)
ER nursing experience, months (mean/SD)	195.4 (146.7)
Current work in ER triage, h/month (mean/SD)	45.9 (20.7)
Experience with computer gaming	
Yes	4
Mean (SD) h/week	2.3 (0.9)
Experience with virtual worlds	
Yes	1
Mean (SD) h/week	1 (–)
Experience with gaming systems	
Yes	4
Mean (SD) h/week	1.4 (0.5)
Experience with cell phone/tablet games	
Yes	6
Mean (SD) h/week	3.8 (4.3)

### Questionnaires and scales

Experience of the simulation task was assessed with questions rated on Likert scales using open-ended questions, such as: “What was your experience like?”, “What would you change?”, and “Do you think this virtual world reflects your real world experience?” An analogue scale assessed subjects’ comfort level using the avatar in the simulation task from “0” (*not at all comfortable*) to “100” (*extremely comfortable*).

The NASA task load index (NASA TLX) [19–22] was used to obtain information about each subject’s subjective workload during both an average day in the ED, and the simulation task. The NASA TLX is a multi-dimensional scale that provides an overall workload score based on a weighted average of ratings on six subscales (*mental demands* needed to perform a task, *physical demands* of the task, *temporal demands* or feeling a time pressure, self-perceived *success during performance*, amount of *effort* put forth, and *frustration* during performance). Each subscale is rated from 0 to 100, with higher scores indicating higher perceived importance. The TLX has been widely used to assess workload in simulations as well as human–machine environments, such as aircraft cockpits and command, control, and communication workstations [21].

### Simulation task

**Fig. 1:**
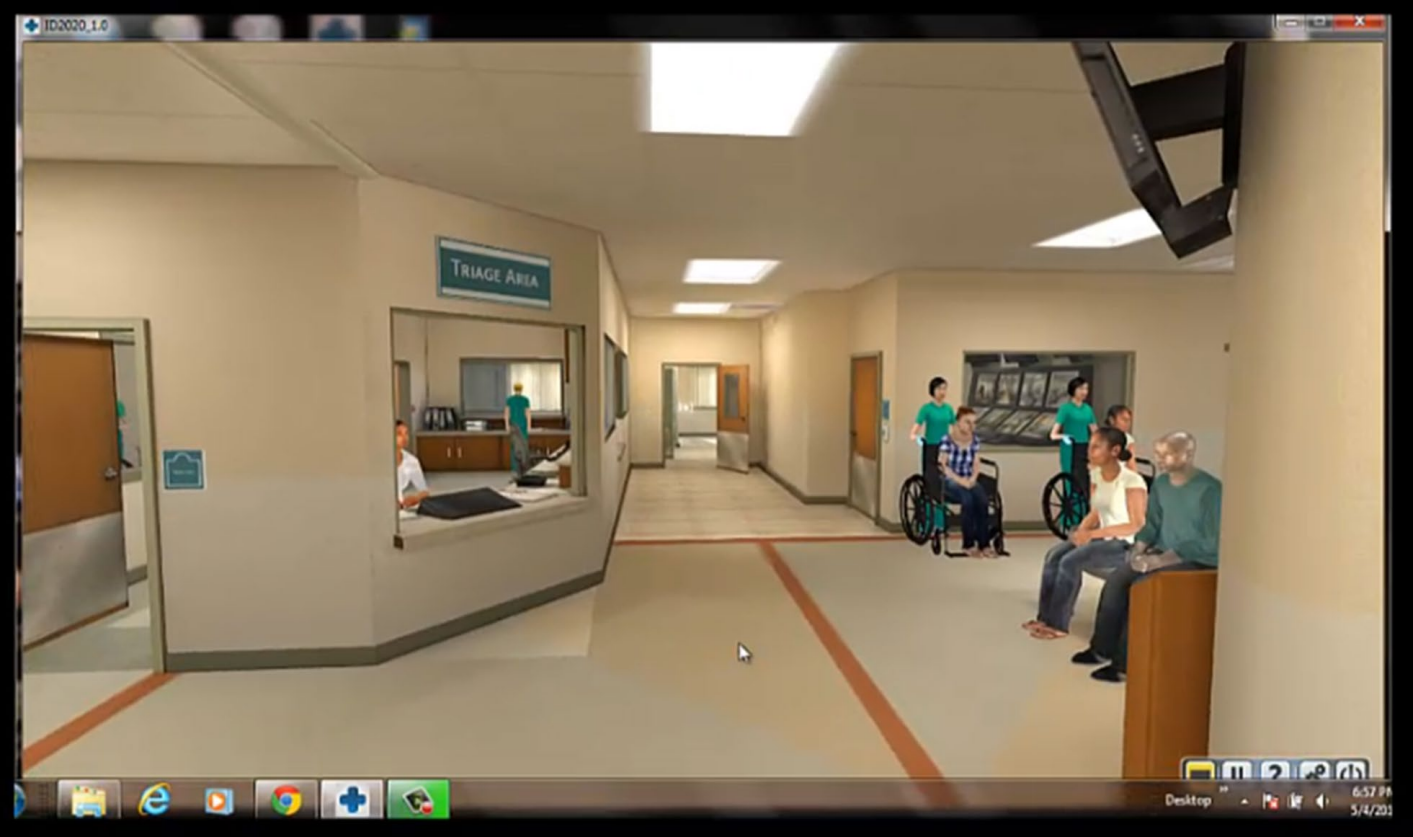
Lobby and reception

**Fig. 2:**
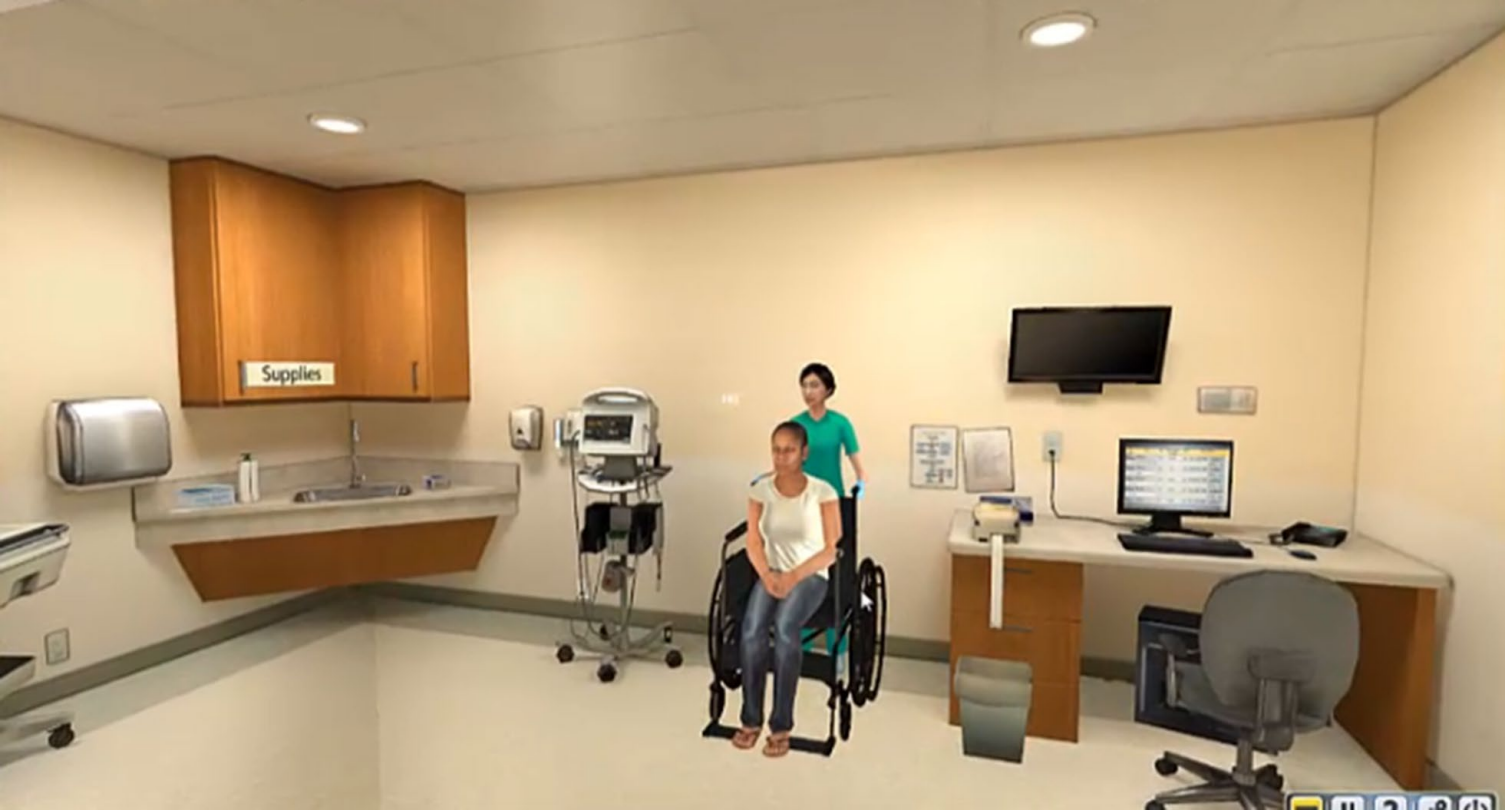
Triage room

**Fig. 3:**
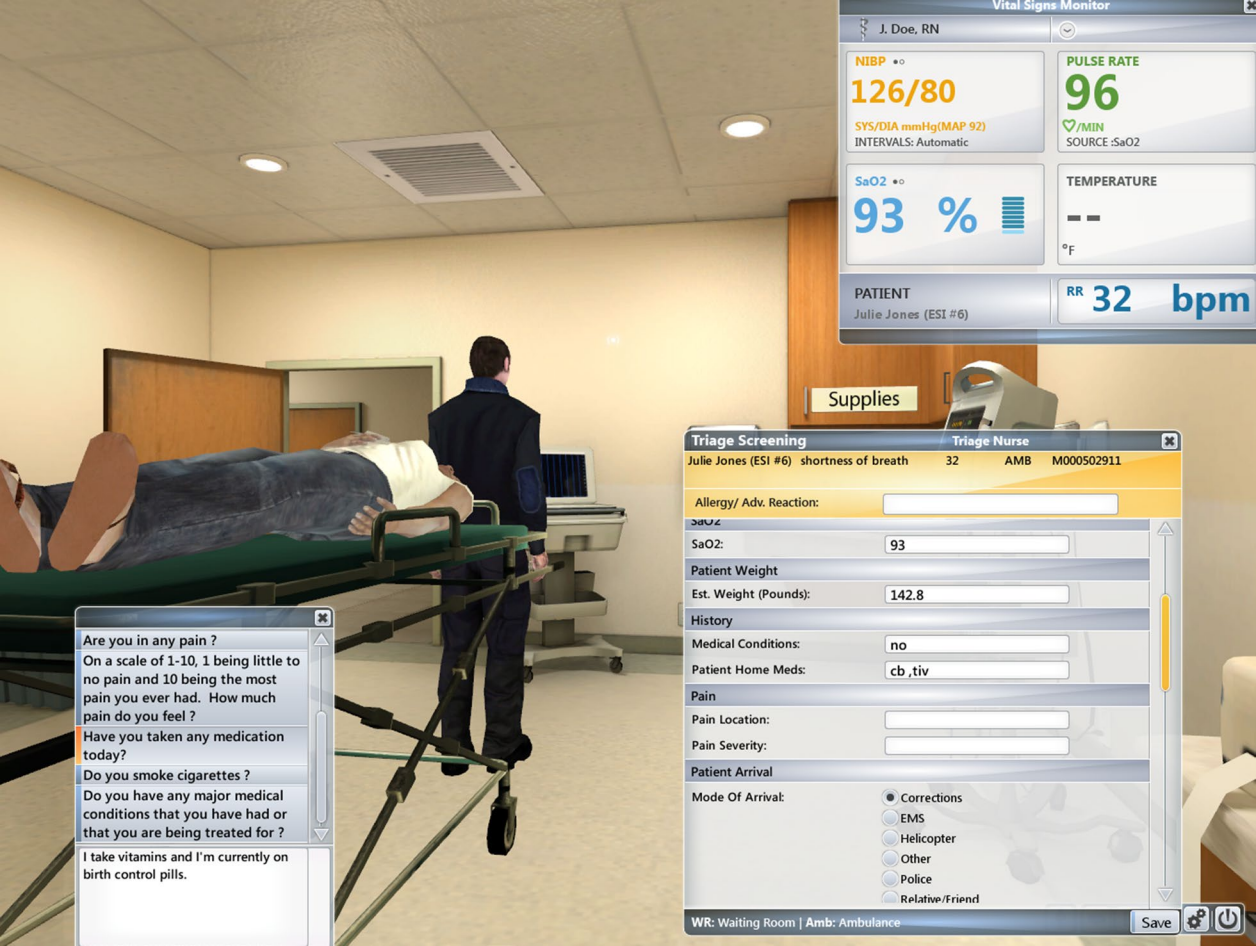
Patient examination with examples of menu options and vital signs

We used CliniSpace [18] to create a 3D computer rendering of the ED of a large urban general hospital (602 inpatient beds, 56,000 general ED and 12,000 psychiatric ED visits/year) that included an ambulance bay, waiting room reception desk, two triage rooms, and connecting hallways (Figs. 1, 2, 3). Standard triage equipment was provided within the environment. The simulation was preloaded with 16 virtual bot (automated) patients. Four of the patients were used to train subjects to navigate in the virtual environment, and the other 12 were used for testing. All patient scenarios represented experience in our ED and were developed using the emergency severity index (ESI) version 4, a triage tool that has been used by ED nursing personnel [23]. Table 2 describes the basic demographics of the 16 patients and their presenting medical conditions.

**Table 2:** Description of simulated patients and their presenting medical issues

Patient avatar	Gender	Ethnicity	Age	Chief complaint	Medical condition	Entry type	Appearance	Time delay
P1	Female	African	27	Abdominal pain	Trauma	Wheelchair	Normal	0:00
P2	Male	African	45	Cough Fever	Pneumonia	Walk in	Pale looking	0:08
P3	Male	Hispanic	65	Fall, head injury	Trauma	Gurney	Static blood on face	0:10
P4	Male	Caucasian	17	High-speed motor vehicle crash	Trauma	Gurney	Static blood on arms	0:10
P5	Female	Caucasian	46	Rash spreading over body	Skin Allergies	Wheelchair	Normal	3:10
P6	Male	Caucasian	58	Difficulty speaking, slurred speech	Stroke	Gurney	Flushed	5:10
P7	Female	African	37	Migraine and vomiting	Trauma	Walk in	Normal	10:05
P8	Male	Hispanic	55	Chest pain moving to left arm	ACS	Walk in	Flushed	10:10
P9	Female	Asian	63	Head injury, assault	Trauma	Walk in	Static blood on face	15:10
P10	Female	African	55	Head Injury	Trauma	Wheelchair	Static blood on face	20:00
P11	Male	Asian	22	Cough, chills and vomiting for 5 h	Pneumonia	Walk in	Pale looking	25:00:00
P12	Male	Caucasian	34	Car crash	Trauma	Gurney	Static blood on arms	30:00:00
trP1	Female	African	32	Shortness of breath	ACS	Gurney	Normal	5:00
trP2	Male	Caucasian	60	Right foot pain	ACS	Wheelchair	Normal	8:00
trP3	Female	Asian	52	Possible urinary tract infection	Pneumonia	Walk in	Pale looking	10:00
trP4	Male	Hispanic	54	Chest pain	ACS	Gurney	Normal	10:00

P, patient; trP, training patient; Time Delay refers to when a patient was presented in the virtual scenario; other variables were also predetermined, including blood pressure, pulse, temperature, respiratory rate, oxygen level, electrocardiogram data, and radio communication notes (these data are available as supplementary data from the authors)

### Procedure

The 3-h study consisted of orientation, testing, and debriefing phases. For the orientation phase, each subject was seated in front of a computer screen equipped with a mouse and keyboard and displaying the virtual triage room in order to learn navigating, interacting, and using objects in the simulation. To avoid potential novelty effects during testing, each task had to be satisfactorily completed before the subject could move on to the next task.

During the testing phase, which followed a 3-min break, subjects seated at the computer manipulated an avatar using arrow keys, beginning at the reception desk (Fig. 1) and navigating to the triage room of the subject’s choice (Fig. 2). The subject’s view was from the avatar’s perspective. Subjects were instructed to triage patients in the simulation just as they would in real life, in the order in which they usually prioritized patients, and to continue the triage process until instructed to stop. The order and timing of new patients presented to subjects remained consistent, but subjects decided which patient was seen next based on their assessment of priority. The simulation ended after each subject had triaged six patients. As is typical of triage in the ED, nurses worked by themselves rather than in groups.

Once in the triage room, the subject directed her avatar to open the triage tracking list and choose the next patient. Two patients appeared in the computer window, and subjects called in the patient they wanted to triage first. With each patient triaged, more patients were added to the tracker. Triage included actions such as hand washing, donning and then disposing of personal protective equipment, obtaining vital signs, and taking a focused history to decide patient disposition (see Additional file 1: Table S1, for a full list of these actions). Subjects could obtain information by selecting questions from a drop-down list and reading the patient’s reply. When a disposition was decided, the subject moved on to the next patient.

### Data analysis

The simulation software generated “transactions” (Additional file 1: Table S1) corresponding to an action performed by the subject (e.g., putting on gloves, reading a blood pressure value) or a change in patient status (e.g., appearance or blood pressure). These transactions were then used to derive non-standardized variables that were used for further analyses (see Additional file 1: Table S2, for more information on non-standardized variables). Because many of the variables were likely to be correlated both with factors dependent on the subject (e.g., triage skills, keyboard literacy, clinical experience), as well as on the patient (e.g., urgency of triage, complexity of the case), a standardized list of variables was constructed by calculating first the z-scores for each subject-patient data point for that variable, and then the mean of the z-scores for each subject based on the patients the subject worked with during the simulation task (Additional file 1: Table S3). This process was used to reduce the effect of differences in patient variables, so that the remaining differences were more likely to be explained by differences in performance on the simulation, while allowing us to assess the accuracy of triage in assessing patient priority and time spent in triaging each patient.

For some variables, being on the negative or positive side of the z-score spectrum could be reasonably associated with a desirable versus non-desirable situation (e.g., it is more desirable for patients to be triaged faster, while it is not more desirable to prefer a particular triage room if there are no differences between the rooms). For this reason, the standardized variables studied were also differentiated on the basis of being desirable (D) or not desirable (nD) (Additional file 1: Table S3).

Data from questionnaires and scales were analyzed with descriptive statistics and paired t-tests using SPSS v. 21. Microsoft Excel was used to compute a correlation matrix for all standardized variables and all simulation data. The matrix was studied for strong positive (>0.75) and negative (<−0.75) correlations between variables. We did not control for multiple comparison because of the small sample size, which reduces the risk of a Type I error [24, 25]. As argued by Nakawaga [25], applying the Bonferroni correction to a small sample with already limited power, reduces power even further, increasing risk for a Type II error to an “unacceptable level” (p. 1045).

## Results

### Subjects’ attitudes and experiences

Responses to the exit questionnaire (Table 3) indicate that the subjects’ attitudes toward the simulation were largely positive. Subjects generally regarded the scenarios as realistic, and when asked specifically whether they thought the virtual world in the simulation task reflected their real world experience, 8 out of 10 subjects answered “yes.” The majority of the subjects noted that the speed of the avatar and of procedures should be increased, but some thought that this was a matter of not having become fully acclimated to the simulation. The mean rating (±SD) of how comfortable subjects felt using the avatar was 46.9 (±19.3), suggesting that participants overall felt moderately comfortable using and maneuvering the avatar, with some subjects feeling distinctly more comfortable than others. The most consistent factor that moderated comfort with the simulation was that interactions with patients and other staff members were via typed questions and answers rather than direct verbal interactions, although the questions were felt to be formulated appropriately.

**Table 3:** Exit questionnaire: attitudes toward the virtual simulation task

1.	During this exercise, to what extent did you feel “immersed” in responding to the simulation exercises?	Not at all 0%	Some of the time 30%	Not sure 0%	Much of the time 40%	All of the time 30%
2.	How easy or difficult was it to learn to take the role of an RN in these simulation exercises (control the avatar)?	Very difficult 0%	Somewhat difficult 40%	Difficult 10%	Somewhat easy 40%	Very easy 10%
3.	Did you experience any technical difficulties when you were working through the simulation exercises today?	None 0%	Infrequently 50%	Several times 30%	Much of the time 20%	Almost all of the time 0%
4.	Prior to today’s exercises, how confident did you feel about your ability to respond to emergency department patients?	Not confident 0%	Somewhat confident 10%	Confident 10%	Very confident 40%	Extremely confident 40%
5.	After completing the simulation exercises today, how confident do you feel about your ability to respond to emergency department patients?	Not confident 0%	Somewhat confident 20%	Confident 0%	Very confident 50%	Extremely confident 30%
6.	How useful do you think these simulation exercises would be for learning the clinical skills necessary to treat patients in an emergency department setting?	Not useful 20%	Somewhat useful 10%	Useful 20%	Very useful 30%	Extremely useful 20%
7.	Did this study change your feelings/attitudes in any way about working as a member or leader of an emergency department Team?	Yes 10%	No 90%			

Paired t-tests on raw NASA TLX subscale scores comparing perceived workload on an average work day with perceived workload on the simulation revealed a significant difference only for physical demand (46.5 ± 24.8 versus 14.5 ± 20.2; p = 0.02), suggesting, as would be expected, that subjects perceived their actual triage work to be more physically strenuous than the simulation task. However, after weighting the scores according to standard procedures (adjusted rating) there were no significant differences across this scale, any of the other scales (mental demand, temporal demand, performance, effort, frustration), or total workload score, indicating that the subjects’ subjective workload demands during the simulation task were equivalent to their subjective workload during a regular work day (Table 4).

**Table 4:** Comparison of mean (and standard deviation) NASA results for an average day at work and for the simulation task

	Average day at work	During simulation task	Statistics
Raw rating			
Mental demand	67.0 (20.8)	77.5 (16.9)	t(9) = −1.64, p = 0.13
Physical demand	46.5 (24.8)	14.5 (20.2)	*t(9)* *=* *2.82, p* *=* *0.02*
Temporal demand	74.0 (14.5)	65.0 (20.5)	t(9) = 1.33, p = 0.21
Performance	32.0 (22.6)	51.5 (17.6)	*t(9)* = −*1.84, p* = *0.09*
Effort	61.0 (23.5)	59.0 (18.9)	t(9) = 0.22, p = 0.83
Frustration	70.0 (21.1)	63.0 (24.7)	t(9) = 0.95, p = 0.36
Adjusted rating			
Mental demand	199.0 (112.0)	235.5 (67.3)	t(9) = −0.85, p = 0.41
Physical demand	40.0 (100.2)	7.0 (22.1)	t(9) = 0.98, p = 0.34
Temporal demand	248.5 (98.6)	232.0 (131.7)	t(9) = 0.29, p = 0.77
Performance	95.0 (74.9)	157.5 (72.3)	t(9) = −1.76, p = 0.11
Effort	137.0 (84.3)	100.5 (36.3)	t(9) = 1.18, p = 0.26
Frustration	179.5 (97.2)	204.0 (153.2)	t(9) = −0.40, p = 0.69
Total weighted rating	59.3 (12.1)	62.4 (13.1)	t(9) = −0.66, p = 0.52

All analyses are paired t tests; significant differences are italics; trend differences are in italics

### Simulation task

The average time to triage a simulated patient was 7:44 ± 2:18 min (range 1:45–13:48 min). Paralleling experience in most settings, there was inter-subject variability on most measures: z-scores for the average time each subject worked on a simulated patient, to control for complexity of patients, showed that subject 2 was fastest at triaging patients in the simulation, while subjects 1 and 8 were slowest (Table 5). However, removing from the analyses patients who were only triaged once eliminated significant differences in triage time between patients. As seen in Table 6, subjects 2 and 10 had more negative z-scores, and subject 1 had more positive z-scores, than the rest of the group, but the differences were not statistically significant. On non-standardized variables (Additional file 1: Tables S4, S5), subjects 2 and 10 had more desirable results, while desirable results were less frequent for subjects 1, 7, and 8. Tables 5 and 6 indicate that subjects were consistent in assigning priority to simulated patients.

**Table 5:** Durations of the time periods subjects (S) worked actively with patients (P) and z-scores

	S1	S2	S3	S4	S5	S6	S7	S8	S9	S10	P mean z-scores
P1			0:06:53 (−0.37)	0:09:11 (0.63)		0:06:16 (−0.64)			0:08:54 (0.51)		0.03
P2		0:04:14 (−1.52)									−1.52
P3	0:07:35 (−0.07)	0:04:52 (−1.25)	0:05:38 (−0.92)	0:11:47 (1.76)	0:09:26 (0.74)	0:06:13 (−0.66)	0:09:51 (0.92)	0:11:36 (1.68)	0:10:03 (1.01)	0:06:13 (−0.66)	0.25
P4	0:08:59 (0.54)	0:04:34 (−1.38)	0:07:35 (−0.07)		0:08:56 (0.52)	0:05:31 (−0.97)	0:08:38 (0.39)	0:08:31 (0.34)	0:08:54 (0.51)	0:08:47 (0.45)	0.04
P5										0:11:05 (1.45)	1.45
P6	0:13:48 (2.64)	0:01:45 (−2.60)	0:07:06 (−0.28)		0:06:58 (−0.34)	0:06:03 (−0.73)	0:06:03 (−0.73)	0:07:25 (−0.14)	0:07:18 (−0.19)		−0.30
P7			0:06:34 (−0.51)								−0.51
P8	0:08:51 (0.48)	0:05:00 (−1.19)	0:07:06 (−0.28)	0:09:36 (0.81)	0:07:23 (−0.15)	0:05:33 (−0.95)	0:09:34 (0.79)	0:09:46 (0.88)	0:05:20 (−1.05)		−0.07
P9		0:04:36 (−1.36)		0:07:15 (−0.21)	0:09:45 (0.87)		0:12:24 (2.03)	0:10:23 (1.15)		0:07:31 (−0.10)	0.40
P10				0:08:09 (0.18)	0:07:08 (−0.26)	0:04:55 (−1.23)	0:10:01 (0.99)			0:06:36 (−0.50)	−0.16
P11											
P12				0:06:54 (−0.36)				0:10:41 (1.28)	0:05:59 (−0.76)	0:05:12 (−1.10)	−0.24
S mean z-scores	0.90	−1.55	−0.40	0.47	0.23	−0.86	0.73	0.86	0.00	−0.07	

Duration is in hour, minutes and seconds [hh:mm:ss]; z-scores are in parentheses. For nominal variables (e.g. in what room the patient would be examined or where he would be triaged to), the higher the z-score, the more a specific option was chosen (e.g. the more a specific triage outcome was chosen)

**Table 6:** Subject specific z-scores for standardized variables

		S1	S2	S3	S4	S5	S6	S7	S8	S9	S10
Patient waiting time	D	0.54	−0.79*	−0.16	−0.10	0.11	−0.47	0.82ʃ	0.36	−0.09	−0.21
Patient call order	nD	0.28ʃ	0.03	−0.07	−0.14*	0.03	−0.07	0.03	0.03	0.03	−0.14*
Patient triage duration	D	0.65ʃ	−1.13*	−0.60	−0.36	0.17	−0.89	0.53	0.63	0.00	−0.65
Patient active work duration	D	0.90ʃ	−1.55*	−0.40	0.47	0.23	−0.86	0.73	0.86	0.00	−0.07
Delay viewing vitals	D	−0.41	−0.36	0.59	−0.47	−1.34*	0.98ʃ	0.51	0.24	0.62	−0.55
Vitals correct if entered	D	0.37	0.12	−0.15	−0.15	−0.25*	0.00	0.00	−0.25*	0.47ʃ	−0.13
Patient name obtained	D	0.35	−0.43*	−0.24	0.35	0.55ʃ	0.55ʃ	−0.04	−0.43*	−0.24	−0.43*
Patient to common triage dest.	nD	0.53ʃ	−1.07*	0.53ʃ	−0.27	0.53ʃ	0.53ʃ	−0.25	−1.03	0.53ʃ	0.14
Patient to common exam room	nD	0.29ʃ	0.29ʃ	−0.28	−0.60*	0.29ʃ	0.29ʃ	0.29ʃ	0.29ʃ	0.29ʃ	−0.60*
Common EDM priority entered	nD	0.30	0.08	−0.32	1.30ʃ	−0.32	−0.82	0.38	0.12	−0.82*	0.23
Hygienic actions	D	1.05ʃ	−0.70	−0.70	1.05ʃ	0.30	0.55	−0.70	0.05	1.05ʃ	−1.95*
Form actions	D	−0.04	−0.09	0.78ʃ	0.78ʃ	0.73	0.78ʃ	−1.23	−0.37	0.78ʃ	−2.10*
Average		0.40ʃ	−0.47	−0.09	0.15	0.08	0.05	0.09	0.04	0.22	−0.54*
D Average		0.43ʃ	−0.62	−0.11	0.20	0.06	0.08	0.08	0.14	0.32	−0.76*
nD Average		0.35ʃ	−0.17*	−0.03	0.07	0.13	−0.02	0.11	−0.15	0.01	−0.09

For each variable, the lowest (*) and highest (ʃ) differences are indicated. Variables that are considered more desirable are noted as D, and variables where desirability does not come into play are noted nD (e.g., more hygienic actions and shorter waiting times are considered desirable). To be statistically significant a variable requires z < −1.96

### Correlations

The correlation matrix conducted for all standardized variables and all simulation data revealed several strong correlations (r ≥ 0.75 or r ≤ −0.75). Subjects who found the overall workload (NASA total weighted rating) of the simulation task to be low had more previous experience using gaming and/or virtual reality systems (r = −0.80), and more hours playing virtual worlds (r = −0.83). Subjects with more current ED experience reported requiring less mental and physical effort (NASA Effort, raw rating work; r = −0.82) and feeling less frustrated/more secure (NASA Frustration, raw rating work; r = −0.81) at work. Currently working in the ED was associated with feeling more successful in and having higher satisfaction with one’s work performance (NASA Performance, raw rating work; r = −0.76). The more confidence subjects felt in their ability to respond to ED patients (Tables 3, 4), the more successful they thought they would be in accomplishing other work tasks (NASA Performance, adjusted rating work; r = −0.79). Although overall confidence in subjects’ ability to respond to ED patients did not change significantly after performing the simulation task (Tables 3, 4 versus 5; r = 0.92), probably because the level of confidence was already high prior to the simulation, the correlation between confidence and performance became stronger (Tables 3, 5 and NASA Performance, adjusted rating work; r = −0.90), suggesting a positive effect of having completed the simulation task.

With respect to performance during the simulation task, simulated patients of subjects with more real-life triage experience spent less time in the waiting room (r = −0.77). Subjects who reported feeling secure and gratified, and less stressed and irritated at their daily job on the NASA Frustration subscale were found to be more likely to enter correct data (e.g., vital signs) during the simulation task (r = 0.78). Greater confidence of subjects in their ability to respond to ED patients (Tables 3, 4) was associated with a higher likelihood of adhering to hand washing and personal protective equipment protocols prior to interacting with the simulated patient (r = 0.81).

A further parallel with actual work flow was that subjects who reported more confidence responding to ED patients (Tables 3, 4) reported less time pressure while doing the stimulation (NASA temporal demand, raw rating simulation; r = −0.81). The less time pressure subjects felt during the simulation (NASA Temporal Demand), the more time elapsed between calling a patient to the exam room and obtaining vital signs (r = −0.90). Vital signs were entered into the chart more accurately by subjects who perceived the simulation to require more mental and perceptual activity (NASA Mental Demand, raw rating simulation; r = −0.75).

## Discussion

The purpose of this preliminary study was to address the validity and feasibility of a newer multi-user virtual reality platform as a proxy for staff behavior in the ED. As can be seen in Tables 5 and 6, in addition to measuring the process of triage (e.g., PPE, interactions with patients), the order in which patients were called and the time spent with each patient was assessed. We were therefore able to evaluate subjects’ ability to prioritize triage patients according to standard principles and procedures. These data, along with a degree of intersubject variability in performance within an expectable range, suggest that virtual reality triage can serve as a valid model of actual ED triage that could facilitate the study of the impact of stresses such as disasters on staff functioning before these events actually occur. As would be expected of a realistic model, more real-life experience working in an ED triage setting was associated with feeling a lower level of workload (e.g., less frustration and less temporal demand) and with better outcomes during the simulation task (e.g., less waiting time for simulation patients). Additionally, feeling less stressed in their daily work and more confident in responding to ED patients was associated with better outcomes during the simulation task (e.g., entering exact data, washing hands, and using personal protective equipment).

In the present study, subjects perceived similar workloads (assessed with the NASA TLX) during their daily work as they did during the computer simulation task. Even though the physical effort of the simulation was, as expected, less than that required in the workplace, subjects reported similar mental demands, and the relationship between time spent in a simulated task and the sense of time pressure while performing it, was similar to the perceived relationship in the workplace. Although familiarity with virtual reality predicted more comfort with the simulation, as has been reported with other platforms [16], self-perceived success and satisfaction with the task, amount of effort put forth, and frustration during the simulated task, were correlated with similar experiences in real-world triage. The impression of a valid relationship between simulated and actual ED experiences is strengthened by 8 of 10 subjects indicating that the virtual world in the simulation task reflected their real world experience.

Some elements of the simulation model should be modified in future work. Subjects felt that the speed and maneuverability of the avatar could be faster. This could be accomplished with greater computing power and enhancing parameters of avatar movement. More extensive training prior to starting the simulation task might address the concern of some subjects that they did not feel fully acclimated to the simulation when testing began. The primary shortcoming of the model involved obtaining patient data through written rather than spoken interactions. Similar concerns have been noted by others when using virtual reality models [18]. Having an experimenter in another room read scripted patient responses to produce a “virtualized” verbal interaction is one cost-effective approach to improving patient-subject interactions. Future models should also address the diversity of clinicians in the ED, the hierarchy of their skills, delegation to other providers, prioritization of tasks, and provider tasks such as teaching and administrative work, or the presence of trainees, who generally slow patient throughput [10]. We are currently modifying the platform to allow us to study interactions of groups of subjects as well as more robust graphics, in a manner that might be useful to institutions that lack computers with sufficient graphics capability or that have firewalls that make accessing servers and downloading more robust programs difficult.

Training with a simulator can improve patient throughput by medical students during simulated triage of a disaster [26], but the impact on actual triage has not been addressed. Computerized simulations have been used to provide information about matching staffing with individual responsibilities and patient flow to reduce length of stay [27], and to improve ED throughput and system performance [1]. One study showed that an actual ED intervention that had been modeled in a simulation resulted in decreased length of stay and decreased the number of patients who stayed longer than 6 h [28]. With only a few exceptions, however, extensive use of physical simulations in medical settings have not resulted in evidence of substantially improved outcomes [9, 29], and the validity of ED responses to disaster that were adapted based on an initial simulation has not been studied.

The challenges of recruiting full-time nursing staff to a time-consuming study limited our sample size in this preliminary study as well as our ability to enroll a random sample of triage nurses, but we thought that the results would be more informative than if the subjects were retired nursing staff or students. Many other studies have utilized small samples, and we were able to collect and analyze extensive data on efficiency and workload management, with statistically and clinically meaningful results. The majority of subjects thought that the exercise was a realistic model of their actual work despite the reality of the scenarios being reduced by dialogue boxes rather than live speech, and performance on the scenarios paralleled reports of actual workplace experience. The validity of this model of ED triage is strengthened by obtaining extensive data on performance on this discrete aspect of emergency work, but generalization of the results to all ED activities is limited, although most nursing staff members who perform triage also perform other ED functions. All subjects were female, which could limit application of the findings to male triage nurses. However, we are not aware of any data indicating gender-specific (or ethnicity-specific) approaches to triage. Obviously, replication of the principles addressed in our study with a larger sample of staff performing more diverse functions would be desirable. The potential usefulness of a research platform does not necessarily translate to effectiveness for training, which requires different functional parameters.

In order to use a practical and cost-effective virtual reality model to study the impact of stresses on ED personnel on ED operations, it is necessary to demonstrate its reliability and validity in representing everyday ED operations that would be expected to be impacted by those stresses. These preliminary results suggest that one essential ED function can be adequately represented in a virtual reality model. If the applicability of the model to triage is confirmed by a larger study of additional ED functions, the model could be used to investigate how ED staff reacts to a variety of stresses and interventions that might optimize their adaptation.

## Conclusions

The prospect of an increasing number of natural and man-made disasters that stress the ED, and particularly its staff, makes it imperative that protocols be developed that will facilitate safe and efficient functioning in the face of events that have not been encountered by many institutions. Computerized simulations can be cost-effective approaches to testing new methods of addressing mass events such as epidemics, if they prove to be valid proxies of actual behavior in the ED. Our results suggest that a virtual reality simulation of an actual ED setting can serve as such a platform.

## Additional file

**Additional file 1.** Additional tables.

## Abbreviations

ED: emergency department
ESI: emergency severity index
NASA TLX: NASA task load index
