# Supplementary material for: A preliminary study of a novel emergency department nursing triage simulation for research applications
Source: BMC Res Notes. 2017 Jan 3;10:15. doi: 10.1186/s13104-016-2337-3 (PMC5217538; doi:10.1186/s13104-016-2337-3)
Supplement: Supplementary file 1 — Additional file 1. Additional tables. [file 13104_2016_2337_MOESM1_ESM.docx]

**SUPPLEMENTARY DATA**

| Table s1: Transactions Recorded in the Simulation Task | | |
| --- | --- | --- |
| Transaction | | Description |
|  | *Transactions in relation to a patient* |  |
| pt1 | TS FORM | Subject registered data in a form |
| pt2 | BP Cuff | Subject attached or removed a blood pressure cuff |
| pt3 | Temperature Sensor | Subject attached or removed a temperature sensor |
| pt4 | Pulse Oximeter | Subject attached or removed a pulse oximeter |
| pt5 | Respiration Sensor | Subject attached or removed a respiration sensor |
| pt6 | View Vital Signs | Subject viewed vital signs |
| pt7 | Get an EKG report | Subject performed an EKG |
| pt8 | Patient's Temperature | The patient’s temperature at the time an action is performed by the subject |
| pt9 | Patient's Blood Pressure (mm/hg) | The patient’s blood pressure at the time an action is performed by the subject |
| pt10 | Patient's Pulse Rate(beats per min) | The patient’s heart rate at the time an action is performed by the subject |
| pt11 | Patient's Respiratory Rate | The patient’s respiratory rate at the time an action is performed by the subject |
| pt12 | Patient's SaO2-Oxygen Saturation | The patient’s oxygen saturation at the time an action is performed by the subject |
| pt13 | Patient Roster Nurse Comment | Subject registers a comment about a patient on the roster |
| pt14 | Patient Roster :User Called/Sent Patient to | Subject sends the patient to some destination |
| pt15 | Patient Menu : User Asked a Question to Patient | Subject asks the patient a question |
| pt16 | Patient Menu : User Transferred Patient | Subject transferred the patient |
| pt17 | Patient spawned | Time patient appears in scenario |
|  | *Transactions dependent on subject alone* |  |
| st1 | Simulation Started | Start time of the simulation |
| st2 | Simulation Ended | End time of the simulation |
| st3 | Disconnected | Time subject exits simulation software |
| st4 | Glove box : user wore gloves | Subject put on gloves |
| st5 | Trash can : user discarded 'mask/glove(s)' in the trash can | Subject discarded mask or gloves in the trash can |
| st6 | Hand Sanitizer : user sanitized hands | Subject sanitized hands |
| st7 | Sink : user washed hands in the sink | Subject washed hands in the sink |
| st8 | Mask box : user wore mask | Subject put on a protective mask |

| Table s2: Derived non-standardized simulation variables | | |
| --- | --- | --- |
| Variable | | Description |
| V1 | Duration of simulation [h:mm:ss] | Calculated per subject by subtracting the start time (st1) of the simulation session from the end time (st2) |
| V2 | Time of first patient action | Amount of time elapsed between the first patient-action performed by a subject (any of the pt-transactions) and the start of a simulation (st1), calculated per subject over all patients (V2s) and per subject per patient (V2sp) |
| V3 | Time of last patient action | Amount of time elapsed between the last patient-action performed by a specific subject (any of the pt-transactions) and the start of a simulation (st1), calculated per subject over all patients (V3s) and per subject per patient (V3sp) |
| V4 | N patients spawned | Number of patients that appeared in the simulation (st4) |
| V5 | N patients called | Number of patients called by subject during the simulation (pt14) |
| V6 | N patients referred | Number of patients referred by subject (pt16) |
| V7 | N non patient actions | Total number of st-transactions, calculated per subject and per patient (V7sp), over all patients per subject (V7s), and over all subjects per patient (V7p) |
| V8 | N non patient form actions | Total number of registered data-elements (pt1) per subject not attributable to a patient |
| V9 | N hygienic actions | Total number of transactions of type st4, st5, st6, st7, and st8 per subject |
| V10 | N patient actions | Total number of pt-transactions, calculated per subject and per patient (V10sp), over all patients per subject (V7s), and over all subjects per patient (V7p) |
| V11 | Average patient action duration | = (V3-V2)/V10, for all versions of V2, V3, and V10 and corresponding averages |
| V12 | Average patient triage duration | = V3 - V2, calculated for each patient for each subject, with averages per patient and per subject |
| V13 | N Asked name | Number of times the subject asked the name of the patient |
| V14 | % vitals correct if taken | Calculated by dividing the number of correctly registered vitals (subset of pt1) – as compared to the vitals displayed by the simulation to the subject (pt6) – by the number of times a subject registered vital information |
| V15 | N hand washings | Number of times the subject washed his hands (st7) |
| V16 | N hand sanitizings | Number of times the subject sanitized his hands (st6) |
| V17 | Patient waiting time | Time lapse between the patient entering the scenario (pt17) and the subject called the patient (pt14) |
| V18 | Patient call order | Order in which the subject selected (pt14) a specific patient for triage |
| V19 | Patient active work duration | Time lapse between the first action the subject performed on the patient other than calling him (any pt-transaction except pt14 and pt17) and the last such action |
| V20 | Delay viewing vitals | Time lapse between the time vitals data are available (pt8 through pt12) and the test subject looks at it (pt6) |
| V21 | Posterior probability of the triage destination the patient was sent to | calculated by dividing the frequency by which a specific triage destination is selected (pt16) by the number of destination selections made by all subjects. |
| V22 | Posterior probability of the selected examination room the patient was sent to | calculated by dividing the frequency by which the room is selected (pt14) by the number of room selections made by all subjects. |
| V23 | Posterior probability of the EDM priority entered for the patient | calculated by dividing the frequency by which a specific EDM priority is registered (pt1) by the number of EDM priority assignments made by all subjects. |

| Table s3: Standardized variables studied with applicability of desirability (D/nD) | | | |
| --- | --- | --- | --- |
| Variable | Description | Desirability | More desirable values |
| sV8 | Non patient form actions | D | Less non patient form actions |
| sV9 | Hygienic actions | D | More hygienic actions performed |
| sV12 | Patient triage duration | D | Shorter triage duration |
| sV13 | Patient name obtained | D | Patient name obtained |
| sV14 | Vitals correct if entered | D | Accurate registration of vitals |
| sV17 | Patient waiting time | D | Shorter waiting time |
| sV18 | Patient call order | nD |  |
| sV19 | Patient active work duration | D | Shorter active work duration |
| sV20 | Delay viewing vitals | D | Shorter delay |
| sV21 | Patient to common triage dest. | nD |  |
| sV22 | Patient to common exam room | nD |  |
| sV23 | Common EDM priority entered | nD |  |
| Note: D = Desirable; nD = desirability does not come into play | | | |

| Table s4: Subject results, mean, sample SDs and intervals for non-standardized derived variables | | | | | | | | | | | | | | | | | |
| --- | --- | --- | --- | --- | --- | --- | --- | --- | --- | --- | --- | --- | --- | --- | --- | --- | --- |
|  |  | S1 | S2 | S3 | S4 | S5 | S6 | S7 | S8 | S9 | S10 | Mean | StDEV.S | -2SD | +2SD | 95% confidence interval for population mean | |
| V1 | Duration [h:mm:ss] | 1:08:11 | 0:31:33 | 0:53:50 | 1:04:41 | 0:58:16 | 0:44:19 | 1:20:00 | 1:07:34 | 0:51:11 | 0:58:39 | 0:57:49 | 0:13:38 | 0:30:32 | 1:25:06 | 0:48:04 | 1:07:35 |
| V2 | Time of first patient action [mm:ss] | 01:28 | 01:25 | 02:54 | 01:44 | 01:06 | 01:05 | 03:31ʃ | 01:04 | 01:03 | 02:11 | 01:45 | 00:52 | 00:02 | 03:28 | 0:01:08 | 0:02:22 |
| V3 | Time of last patient action [h:mm:ss] | 1:07:37 | 0:30:56 | 0:49:23 | 1:03:38 | 0:56:53 | 0:42:22 | 1:17:39 | 1:06:02 | 0:49:57 | 0:58:10 | 0:56:16 | 0:13:35 | 0:29:05 | 1:23:26 | 0:46:32 | 1:05:59 |
| V4 | N patients spawned | 12 | 12 | 12 | 12 | 12 | 12 | 12 | 12 | 12 | 12 |  |  |  |  | 0 | 0 |
| V5 | N patients called | 4ʃ | 6 | 7 | 8 | 6 | 7 | 6 | 6 | 6 | 8 | 6.40 | 1.17 | 4.05 | 8.75 | 6 | 7 |
| V6 | N patients referred | 4ʃ | 6 | 6 | 6 | 6 | 6 | 6 | 6 | 6 | 6 | 5.80 | 0.63 | 4.54 | 7.06 | 5 | 6 |
| V7 | N non patient actions | 17 | 25 | 7 | 0 | 4 | 2 | 49 | 28 | 0 | 72* | 20.40 | 24.01 | -27.63 | 68.43 | 3 | 38 |
| V8 | N non patient form actions | 17 | 18 | 0 | 0 | 1 | 0 | 42 | 24 | 0 | 60* | 16.20 | 20.90 | -25.59 | 57.99 | 1 | 31 |
| V9 | N hygienic actions | 0 | 7 | 7 | 0 | 3 | 2 | 7 | 4 | 0 | 12 | 4.20 | 3.99 | -3.79 | 12.19 | 1 | 7 |
| V10 | N patient actions | 231 | 310 | 234 | 294 | 281 | 244 | 251 | 240 | 319 | 300 | 270.40 | 33.91 | 202.58 | 338.22 | 246 | 295 |
| V11 | Average patient action duration [mm:ss] | 00:17 | 00:06 | 00:12 | 00:13 | 00:12 | 00:09 | 00:18 | 00:16 | 00:09 | 00:11 | 00:12 | 00:04 | 00:05 | 00:20 | 00:10 | 00:15 |
| V12 | Average patient triage duration [mm:ss] | 09:48 | 04:10 | 06:49 | 08:49 | 08:16 | 05:45 | 09:25 | 09:44 | 07:45 | 07:34 | 07:48 | 01:50 | 04:09 | 11:28 | 06:30 | 09:07 |
| V13 | N Asked name | 2 | 5 | 4 | 1 | 0 | 0 | 3 | 5 | 4 | 5 | 2.90 | 2.02 | -1.15 | 6.95 | 1 | 4 |
| V14 | % vitals correct if taken | 83.3% | 90.0% | 97.2% | 97.2% | 100.0% |  | 77.8% | 66.7% | 80.6% | 80.6% | 0.86 | 0.11 | 0.64 | 1.08 | 77.5% | 94.4% |
| V15 | N hand washings | 0 | 6 | 5 | 0 | 1 | 0 | 0 | 0 | 0 | 4 | 1.60 | 2.41 | -3.23 | 6.43 | 0 | 3 |
| V16 | N hand sanitizings | 0 | 1 | 2 | 0 | 2 | 0 | 6 | 4 | 0 | 4 | 1.90 | 2.13 | -2.36 | 6.16 | 0 | 3 |
| Tabe s4 displays for the non-standardized variables V1 through V16 individual values for each subject, the mean and standard deviation (SD) for the 10 subjects, the lower (-2SD) and upper (+2SD) thresholds for being considered an outlier within the sample, and the 95% confidence intervals for the population means. Significant outliers within the sample of 10 subjects are indicated with * (favorable direction) or ʃ (unfavorable direction), based on two standard deviations from the mean. Comparing the individual values with the -2SD and +2SD confidence intervals, subjects 2 and 10 as subjects had more favorable results and 1, 7 and 8 had less favorable results than their peers | | | | | | | | | | | | | | | | | |

| Table s5: Ranking of subjects over standardized variables with favorability direction | | | | | | | | | | |
| --- | --- | --- | --- | --- | --- | --- | --- | --- | --- | --- |
|  | S1 | S2 | S3 | S4 | S5 | S6 | S7 | S8 | S9 | S10 |
| Patient waiting time | 9 | 1ʃ | 4 | 5 | 7 | 2 | 10* | 8 | 6 | 3 |
| Patient triage duration | 10* | 1ʃ | 4 | 5 | 7 | 2 | 8 | 9 | 6 | 3 |
| Patient active work duration | 10* | 1ʃ | 3 | 7 | 6 | 2 | 8 | 9 | 5 | 4 |
| Delay viewing vitals | 4 | 5 | 8 | 3 | 1ʃ | 10* | 7 | 6 | 9 | 2 |
| Vitals correct if entered | 9 | 8 | 3 | 3 | 1ʃ | 7 | 6 | 2 | 10* | 5 |
| Patient name obtained | 7 | 1 | 4 | 7 | 9* | 9* | 6 | 1ʃ | 4 | 1ʃ |
| Hygienic actions | 8* | 2 | 2 | 8* | 6 | 7 | 2 | 5 | 8* | 1ʃ |
| Form actions | 5 | 4 | 7* | 7* | 6 | 7* | 2 | 3 | 7* | 1ʃ |
| Sum of ranks | 62* | 23 | 35 | 45 | 43 | 46 | 49 | 43 | 55 | 20ʃ |
| Rank of sums | 10* | 2 | 3 | 6 | 4 | 7 | 8 | 4 | 9 | 1ʃ |

Table s5 provides a view on the ranking of the subjects on each of the standardized variables that carry a desirability notion, as well as on their overall ranking taking all such variables into account. Results indicated by * reflect the near-significantly less desirable nature of the corresponding values upon which the ranking is based, while ʃ indicates near-significant higher desirability. This overall perspective singles out S1 as the subject that performed in the least favorable way, and S10 in the most favorable way, although results just missed significance
